# 1-Bit Transmission-Type Digital Programmable Coding Metasurface with Multi-Functional Beam-Shaping Capability for Ka-Band Applications

**DOI:** 10.3390/mi14061250

**Published:** 2023-06-14

**Authors:** Aqeel Hussain Naqvi, Duc Anh Pham, Syed Imran Hussain Shah, Sungjoon Lim

**Affiliations:** School of Electrical and Electronics Engineering, Chung-Ang University, Seoul 06974, Republic of Korea; aqeelhnaqvi89@gmail.com (A.H.N.); anhducbk.3i@gmail.com (D.A.P.)

**Keywords:** transmissive metasurface, digital coding metasurface, multi-functional, beam shaping

## Abstract

Digital programmable coding metasurfaces (DPCMs) have recently attracted enormous attention and have been broadly applied, owing to their ability to manipulate electromagnetic (EM) wave behaviours and programmable multi-functionality. Recent DPCM works are divided into reflection and transmission types (R-DPCM and T-DPCM, respectively); however, there are only a few reported T-DPCM works in the millimetre-wave spectrum, owing to the difficulty of realising the large-phase controllable range while maintaining low transmission losses with electronic control components. Consequently, most millimetre-wave T-DPCMs are demonstrated only with limited functions in a single design. Additionally, all these designs use high-cost substrate materials that constrain practical applicability, owing to cost-ineffectiveness. Herein, we propose a 1-bit T-DPCM that simultaneously performs three dynamic beam-shaping functions with a single structure for millimetre-wave applications. The proposed structure is completely constructed using low-cost FR-4 materials, and operation of each meta-cell is manipulated using PIN-diodes, thus driving the achievement of multiple effective dynamic functionalities including dual-beam scanning, multi-beam shaping, and orbital-angular-momentum-mode generation. It should be noted that there are no reported millimetre-wave T-DPCMs demonstrating multi-function design, thus showing a gap in the recent literature of millimetre-wave T-DPCMs. Moreover, cost-effectiveness can be significantly enhanced, owing to the construction of the proposed T-DPCM using only low-cost material.

## 1. Introduction

Recent decades have witnessed remarkable developments in metamaterials, owing to their exotic electromagnetic (EM) behaviours, which have attracted widespread attention from physicists and engineers alike. Meanwhile, the two-dimensional (2D) counterparts of metamaterials, which are well known as metasurfaces, have been intensively investigated for their promising advantages of compactness, low cost, high-level integration, and ease of fabrication. Recently, metasurfaces have found their stands in diverse innovative applications, including reconfigurable radiating performances [1,2,3,4], polarisation converters [5], electromagnetic cloaks [6,7,8], vortex-beam generation/manipulation [9,10,11], holography [12,13,14,15,16], and beam deflectors [17,18,19].

Multiple EM functionalities can be achieved with reconfigurable and programmable (active) metasurfaces, whereas most of the reported passive metasurfaces are able to execute only a single EM function [9]. In 2014, the idea of a digital programmable coding metasurface (DPCM) was proposed to manipulate EM waves using digital stimuli [20]. Unlike traditional metasurfaces that are realised by modulating pattern geometries to achieve transmission meta-cell phase profiles, DPCMs are specified using binary codes in a digital format. One-bit coding metasurfaces, for example, are composed of two separate coding elements denoted by the digits “0” and “1”, which indicate opposing phases of 0° and 180°, respectively [21,22]. Since the proposal of original work in 2014, DPCMs have undergone a period of fast innovation. Although fabrication and design of DPCMs still pose many challenges, especially for millimetre-wave frequencies, DPCMs can ensure considerable benefits for performing various real-time functions using field-programmable gate arrays (FPGAs) to execute different coding sequences [23,24]. As a result, various applications, such as anomalous scattering, radar cross-section reduction, and beam shaping with multiple orbital angular momentum, can be enabled in the microwave, terahertz, and even acoustic frequencies. Furthermore, the concept of the DPCM has been extended further to integrate intelligent systems for improving wave manipulation and achieving more innovative applications [25]. On the other hand, several works have tried to achieve multi-functional controllable metasurfaces (MSs) with all-passive mechanisms [26,27,28] or proposed new ideas for all-passive controlling methods [29]. However, there are limitations to these works, owing to the lack of control freedom, which limits their practical application.

DPCMs can be classified into two types as reflection and transmission type (R-DPCM and T-DPCM). Given the rapid development of wireless communications in the millimetre-wave spectrum for achieving high data rates and minimal latencies [30,31,32,33], considerable efforts have been invested into research on both R-DPCM and T-DPCM in the millimetre-wave band. Meanwhile, PIN-diodes and varactor diodes have been widely used for enabling controllable DPCMs [34,35,36,37,38,39,40,41,42,43]. However, recent studies have focused more on the former technique of R-DPCMs, and only a few works have been reported for T-DPCMs in the millimetre-wave band because of the design challenges associated with realising large controllable ranges of the transmission phase delays while maintaining high transmission efficiencies in this frequency range [37].

To the date, most of the reported T-DPCMs in the millimetre-wave band have demonstrated limited numbers of functions, where each design in this frequency range typically has only one or two functions. Moreover, all of these designs use high-cost, high-performance substrates that constrain their practical applicability, owing to cost ineffectiveness. FR-4 epoxy is well known as a low-cost material that is widely applied in the practical low-frequency applications, owing to its cost-effectiveness and ease of fabrication. Nevertheless, due to its high loss and unstable performance in the high-frequency range, FR-4 is typically not a preferred material for practical millimetre-wave applications. In particular, the majority of reported millimetre-wave DPCMs are proposed and demonstrated with high cost or complicate-fabrication materials such as Duroid substrate, synthesised materials (polymide + quartz + gold + liquid crystal) or (silicon glass + nemantic liquid crystal + polymide), and so on [38,41,43]. As a consequence, extant literature on millimetre-wave T-DPCMs lacks works involving complete construction using only low-cost substrates while still effectively allowing multiple functions with good performances, thus creating a gap between millimetre-wave DPCM and practical applications.

**Figure 1:**
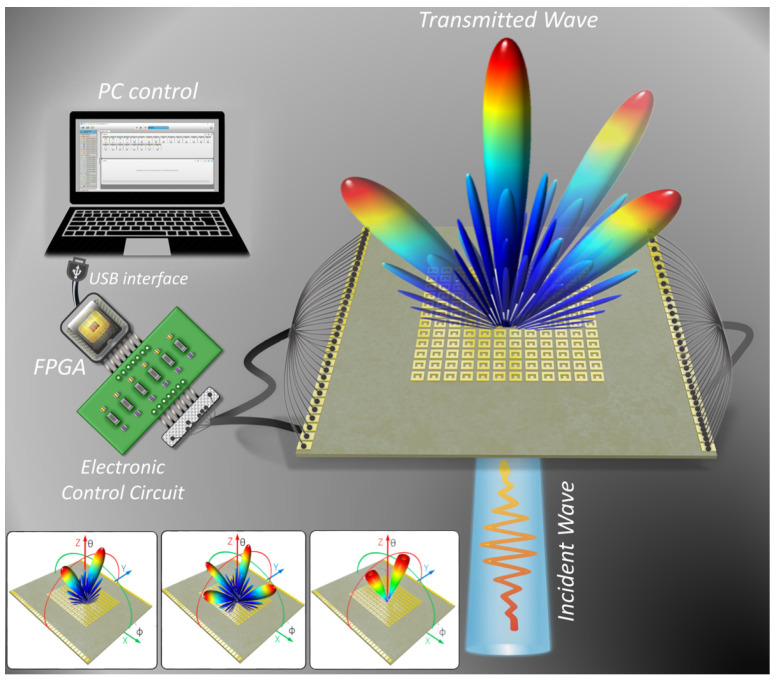
Conceptual illustration of the proposed 1-bit transmission-type digital programmable coding metasurface (T-DPCM) with full-space EM-wave control. The proposed metasurface platform incorporates an FPGA and electronic circuitry to achieve three beam-shaping functions, namely, dual-beam scanning, multi-beam shaping, and OAM-mode dual-vortex-beam generation.

In this study, we experimentally realised a transmission-type DPCM for Ka-band applications with multi-functional beam-shaping capabilities, as depicted in Figure 1. Although the unstable permittivity of low-cost FR-4 material is unavoidable, we can effectively minimise their tentative effects on meta-cell performances by optimising each part of meta-cell design including meta-cell geometrical parameters and biasing network. To accomplish real-time adjustments of the transmission phase code without affecting the polarisations of the incident waves, we employed PIN diodes to independently control each meta-cell. The transmission phase code is 1-bit and comprises the digital values “0” and “1”, which represent 0° and 180° phase, respectively. The proposed design effectively performs multiple dynamic beam-shaping functionalities, including dual-beam steering, multiple beam shaping, and vortex beams with orbital-angular-momentum (OAM) generation by generating different digital coding sequences. It should also be noted that the proposed structure was constructed entirely using the low-cost substrate FR-4, which significantly enhances the cost effectiveness of the design. The proposed T-DPCM is thus expected to be a potential candidate for practical applications in the millimetre-wave spectrum.

## 2. Materials and Methods

### 2.1. Meta-Cell Design and Analysis

**Figure 2:**
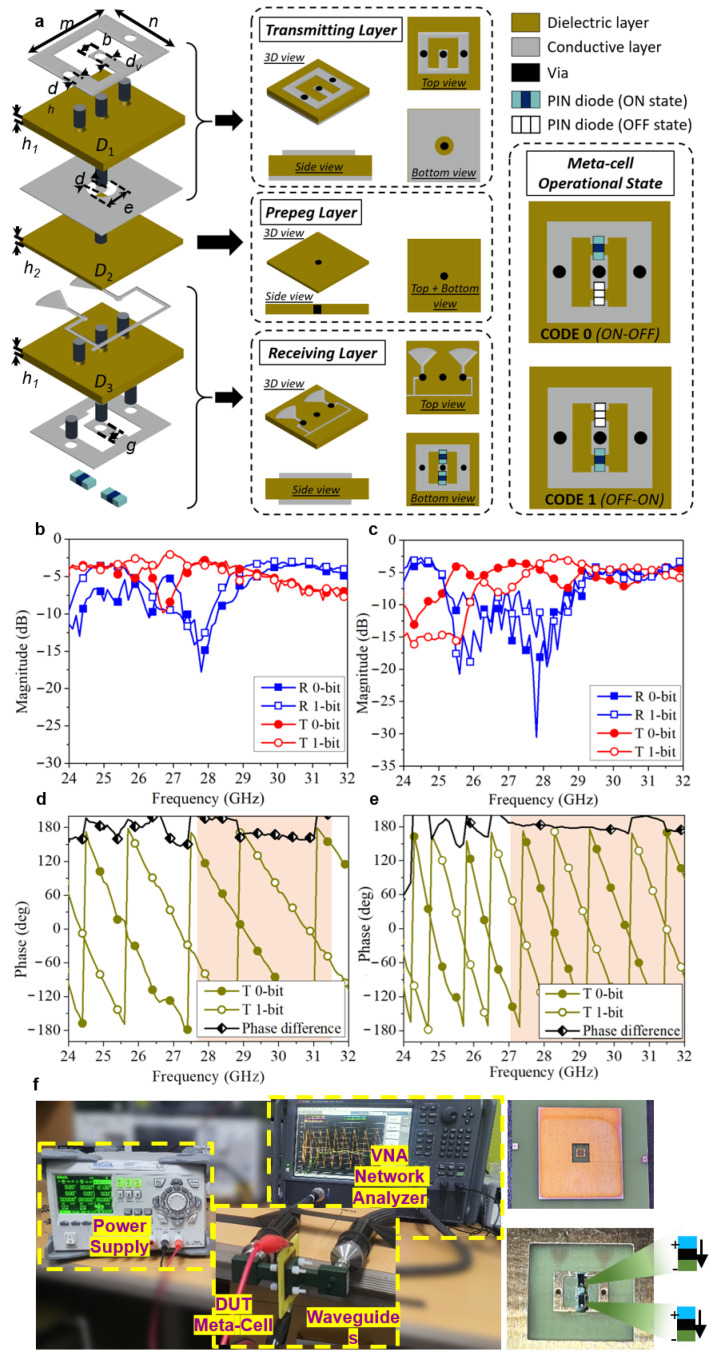
Analytical and experimental investigations of the designed meta-cell. (**a**) Detailed 3D view of the meta-cell of the proposed T-DPCM, including top transmitting layer, adhesive prepeg layer, and bottom receiving layer. (**b**,**c**) Simulated and measured reflection/transmission coefficient magnitudes and (**d**,**e**) phases of the meta-cell for different operational states. (**f**) Meta-cell experimental measurement configuration.

A 1-bit meta-cell working under linear polarisation is the foundational block of the proposed T-DPCM. Figure 2a demonstrates structure of designed meta-cell. Two substrate layers of FR-4 (*ε*_R_ = 4.4, tan*δ* = 0.02) are bonded together with an adhesive prepreg layer (*ε*_R_ = 4.4, tan*δ* = 0.02), and four metallic conductive layers make up the stack. The thickness of dielectric layers (FR4-epoxy) is fixed at 0.5 mm, and an adhesive prepreg layer modelled as a dielectric with the thickness of 0.2 mm. A metallic patch with an O-shaped aperture is used on the bottom of the receiving layer, whereas a metallic patch with a U-shaped slot is used on the top side of the transmitting layer. Two metallic vias are used in the transmitting layer to shorten the metallic patch on the top with the ground on the bottom side of the transmitting layer. Meanwhile, the patches in the transmitting and receiving layers are connected by the via at the centre, as depicted in Figure 2a. Two PIN diodes are integrated with the patches in the receiving layer and switched to control signal flow, thus creating transmission phases of 0° and 180°. Because of its low insertion loss and compact size, we chose the PIN diode MA4GP907 as the active tuneable device. The diode is modelled as a series RL circuit for forward bias (ON state), i.e., *R*_ON_ = 5.2 Ω; *L*_ON_ = 0.05 nH; and as a shunt RC circuit, i.e., *R*_OFF_ = 40 kΩ, *C*_OFF_ = 0.025 pF, with reverse bias for the OFF state. A single DC bias line with open-ended radial stubs is designed to effectively isolate the radio frequency (RF) signals and DC power supply. Each operational state of the designed meta-cell is illustrated in Figure 2a, and the detailed dimensions are provided in Appendix A.

**Figure A2:**
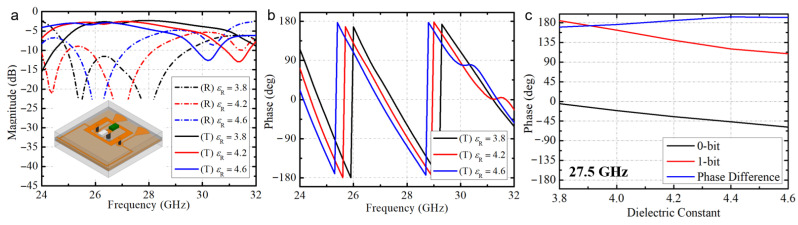
Proposed meta-cell performance with variation of substrate permittivity. (**a**,**b**) Transmission magnitude and phase variations. (**c**) Phase difference variation at 27.5 GHz.

On the basis of minimising possible effects of unstable performance low-cost materials to the final fabricated prototype, at the beginning, we thoroughly investigated different FR-4 substrates provided by a same manufacturer. Since FR-4 is a low-cost substrate, FR-4 provided by the same manufacturer can still have different permittivity and loss. By investigating a series of FR-4 substrate samples from same manufacturer, the variation range of substrate permittivity and loss can be quantitatively profiled. While higher-loss of low-cost material is unavoidable and we have to accept a slightly trade-off of 1–2 dB in transmission coefficient, the geometrical parameters of each meta-cell design and biasing network are optimised to minimise the effects of permittivity variations within previous profiled range. Generally, even if the substrate permittivity is varied, the transmission phase difference between two meta-cell states of 0-bit and 1-bit remain approximately 180° due to the configuration of two diodes in opposite directions, which is around 170° to 190°, as demonstrated in Figure A2 in Appendix A. However, the permittivity variations can significantly affect to transmission magnitude of designed meta-cell. In particular, we investigated the meta-cell design with different geometrical parameters to find out which parameter combinations of meta-cells can give acceptable performances with most of substrate permittivity values within the previous profiled range. Meanwhile, different types of biasing network were investigated to find out which bias technique is least sensitive with substrate permittivity variation. Full-wave analysis showed that biasing network using radial stubs exhibited the less sensitive performance with permittivity variation, and thus biasing network using radial-stubs was chosen for this design.

Owing to the opposite direction of diodes, a 1-bit reconfigurable transmissive meta-cell with perfect binary coding phase (180° phase difference) was achieved at the frequency of 28 GHz. Using the following equation, the quantised phase profile can be profiled as binary digital values [38]:

(1) $$code\left(m,n\right)=\left\{\begin{array}{c} 0,\;0<\varphi \left(m,n\right)<\pi \\ 1,\;\pi <\varphi \left(m,n\right)<2\pi \end{array}\right.$$

As illustrated in Figure 2, a prototype of the meta-cell was simulated and tested in a standard WR28 waveguide configuration; a coax-to-WR28 adaptor and a WR28 straight waveguide section constitute the WR28 waveguide (7.112 mm × 3.556 mm). The original meta-cell is enclosed so as to ensure that the surrounding metallic parts contact the WR28 flanges, thereby ensuring waveguide wall continuity and preventing propagation outward from the meta-cell. On the edge of the substrate, two bias lines and their associated pads are incorporated to appropriately control the diode states. Using conductive silver epoxy, we attached the two MA4GP907 PIN diodes to the fabricated prototype.

The simulated and measured S-parameters of the designed meta-cell are given in Figure 2b–e. In the meta-cell experimental investigation, bias voltages of 0 V and 1.3 V were used for establishing 0-bit and 1-bit states of meta-cells. In particular, the centre via and side vias were supplied with bias voltages of 0 V/1.3 V (for 0-bit state) and 1.3 V/0 V (for 1-bit state). The detail meta-cell information in two operational states is given in Table 1. Specifically, the simulated S-parameters showed minimum insertion losses of 2.9 and 3.3 dB for the 0-bit and 1-bit states, respectively, whereas the measured S-parameters showed minimum insertion losses of 3.4 and 4.5 dB for the 0-bit and 1-bit states, respectively. Additional losses of approximately 1.2 dB were observed during the experiments, which were attributed to the fabrication errors, measurement setup uncertainties, and differences in the FR-4 dielectric substrates. At the design frequency of 28 GHz, the measured phase difference between the two transmission states was 182.6°; hence, the maximum error in the transmission phase was around 23°.

### 2.2. Metasurface Design and Analysis

**Figure 3:**
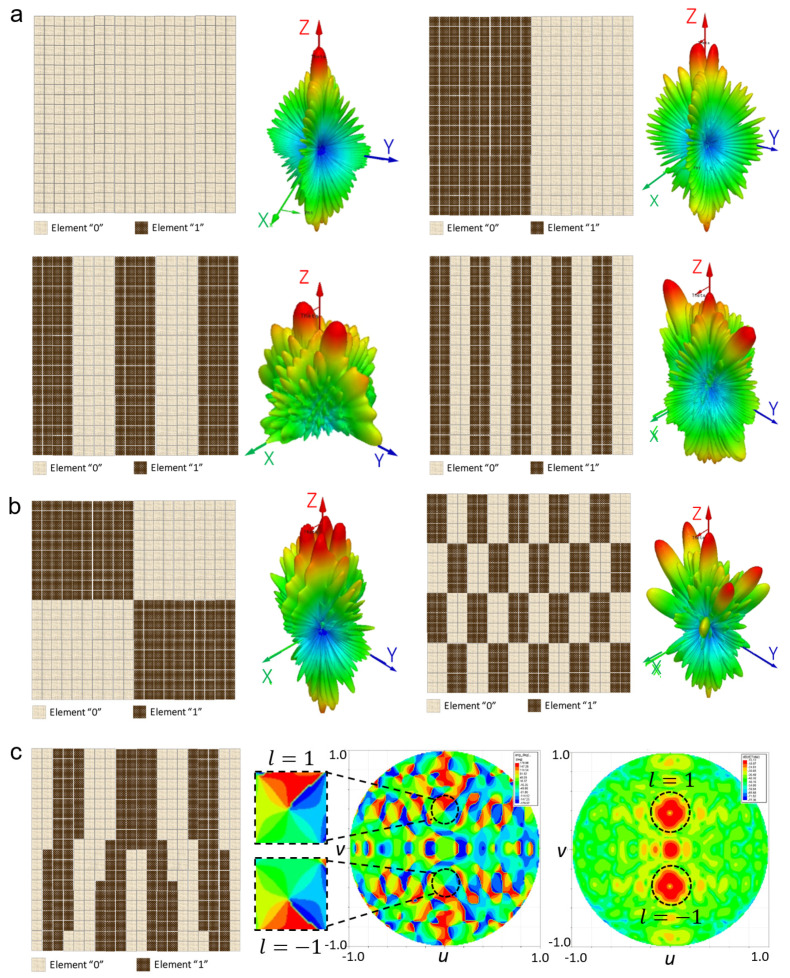
Coding patterns and radiation patterns of multiple functions with the proposed T-DPCM. (**a**) Dual-beam scanning, (**b**) multi-beam generation, and (**c**) OAM-mode dual-vortex-beam generation.

To construct a T-DPCM, the proposed meta-cell was periodically organised in a two-dimensional geometry. Each meta-cell was equipped with two PIN diodes, which were controlled by a separate bias line for each meta-cell. At the design frequency of 28 GHz, a meta-cell with perfect binary coding phase (180° phase difference) was accomplished by regulating both diodes individually. Multiple functionalities were obtained by coding these meta-cells. In this work, we designed a metasurface with 20 × 20 elements to examine the beam-shaping and manipulation performances of the proposed T-DPCM. Three main functions were investigated, namely, dual-beam steering, multi-beam shaping, and OAM-mode dual-vortex-beam generation, as depicted in Figure 3. Numerical analysis and simulations were performed to verify the performance of the proposed T-DPCM.

With free-space boundary conditions, a linear-polarised incident plane wave transmits toward normal of the designed metasurface. The normally incident plane wave is transmitted through the metasurface undeflected in the broadside direction when all elements are encoded as either “0” or “1”, i.e., with coding sequences “000000…/000000…” or “111111…/111111…”, respectively. Various beam-shaping and scattering functions are obtained by coding each meta-cell with a different coding sequence for the entire structure. For instance, the incident plane wave is redirected in two symmetrical directions under the coding sequence “110011…/110011....” Figure 3a depicts several dual-beam patterns under various coding sequences. The mechanisms of these effects can be fully understood by examining the T-DPCM to determine the radiation patterns of specific coding sequences made up of *M* × *N* equal-sized meta-cells. In principle, because beam steering for a metasurface is comparable to a phased array, the far-field function of a metasurface constructed with *M* × *N* meta-cells is represented as follows [38]:

(2) $$F\left(\theta ,\varphi \right)=f_{e}\left(\theta ,\varphi \right)\sum_{m=1}^{M}\sum_{n=1}^{N}exp\left\{-i\left\{\varphi \left(m,n\right)+\frac{2\pi }{\lambda }dsin\theta \left[\left(m-\frac{1}{2}\right)cos\theta +\;\left(n-\frac{1}{2}\right)sin\theta \right]\right\}\right\},$$

where the elevation and azimuthal angles of an arbitrary direction are designated as *θ* and *φ*, respectively; *F*(*θ*, *φ*) refers to the resulting pattern; *f_e_* (θ,*φ*) is the pattern function of an element; and *φ*(*m*, *n*) refers the phase of the meta-cell at position (*m*, *n*). If the number of meta-cells is finite, the function |*F*(*θ*,*φ*)| can reach its maximum only when the elevation angle *θ* and azimuthal angle *φ* fulfil the following conditions:

(3) $$\varphi =\pm \tan^{-1}\frac{\Gamma_{x}}{\Gamma_{y}},\varphi =\pi \pm \tan^{-1}\frac{\Gamma_{x}}{\Gamma_{y}},$$

(4) $$\theta =\sin^{-1}\left(\lambda \sqrt{\frac{1}{\Gamma_{x}^{2}}+\frac{1}{\Gamma_{y}^{2}}}\right),$$

where *Γ_x_* and *Γ_y_* are the coding sequence periodical lengths in the *x* and *y* directions, respectively. According to Equations (3) and (4), if the periodicity is adjusted, the direction of the resulting beam can be steered correspondingly, thus verifying the observed performance of the proposed T-DPCM illustrated in Figure 3a.

Figure 3b demonstrates the proposed T-DPCM operating with the multi-beam shaping function. By applying a chessboard coding pattern [23], the proposed T-DPCM performs multi-beam radiation so that the beam directions and number of scattered beams can be controlled by changing the periodicity of the coding pattern. Two configurations were investigated, demonstrated in Figure 3b, to verify the capability of the proposed structure for multi-beam shaping.

Meanwhile, since the topological charges of the OAM or vortex beams are mutually orthogonal, these beams provide an infinite number of channels in communication systems without extending the frequency bandwidth. To generate an OAM beam with topological charges *l*, the phase of the element (*m*,*n*) is given by

(5) $$\varphi \left(m,n\right)=\varphi_{o}+l\theta_{mn}$$

where *θ_mn_* is the azimuthal angle of the element (*m*,*n*)

(6) $$\theta_{mn}=\tan^{-1}\frac{y}{x}$$

and *φ*_o_ is the initial phase. The phase of the incident plane wave is 0°. The initial phase *φ*_o_ and OAM phase *l_φ_*_o_ make up the phase of the elements. Figure 3c shows the coding scheme and simulated far-field output of the *l* = ±1 OAM beam at 28 GHz. The *l* = ±1 OAM’s doughnut-shaped intensity can be observed clearly, with the phase distributions of both beams being uniform and exhibiting the typical properties of OAM beams.

### 2.3. Software-Controlled Circuit Design

**Figure 4:**
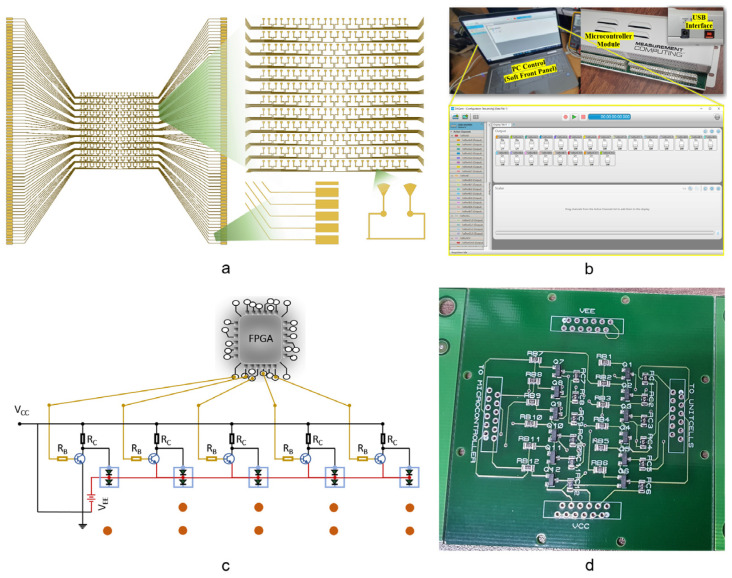
DC bias network design and setup of the controlling modules. (**a**) Design of DC bias network. (**b**) Software control panel. (**c**) Schematic of the bipolar-transistor-based control circuit for two meta-cells. (**d**) Fabricated PCB of the extended interface module of the electronic circuit.

The DC bias network is constructed using individual bias lines, as stated in the meta-cell design section. For the entire structure, the biasing circuit is grouped into two identical sub-arrays, as shown in Figure 4a. As a result, each meta-cell may have one to several bias lines traversing in the proximity of the active patch depending on their placements within a sub-array. These bias lines were tested and proved to have negligible influence on the RF performance, allowing additional bias lines to be tolerated for larger designs.

In general, a programmable MS may be controlled directly by the FPGA output pins; however, the number of FPGA pins is frequently insufficient to operate programmable MSs that need a large number of control inputs. Furthermore, if the MS is directly linked to the FPGA pins, the MS operations will be disrupted if the FPGA controller fails, such as for an FPGA power-down program error or connection break-off. To overcome these two issues, an expanded interface circuit that uses bipolar NPN transistors to accomplish the switching functions was constructed. As a result, we have two control interfaces (USB-DIO96H modules, Measurement Computing Company (MCC), Hungary) in our programmable control configuration. The outputs of the FPGA pins are controlled in real time through a GUI soft front-panel interface, as shown in Figure 4b. The designed buffering printed-circuit board (PCB) is used to connect these control modules to the signal line on the MS structure. We used bipolar NPN junction transistors S8050-G in a SOT-23 package. The values of the base and collector resistors were tuned and optimised; *R*_b_ = 1 kΩ and *R*_c_ = 470 Ω were chosen as the base and collector resistor values, respectively. The designed schematic is shown in Figure 4c. In this circuit, we only show two meta-cells for the purpose of brevity. The final fabricated interface is shown in Figure 4d.

## 3. Experimental Verification and Results

### 3.1. Prototype Realisation

**Figure 5:**
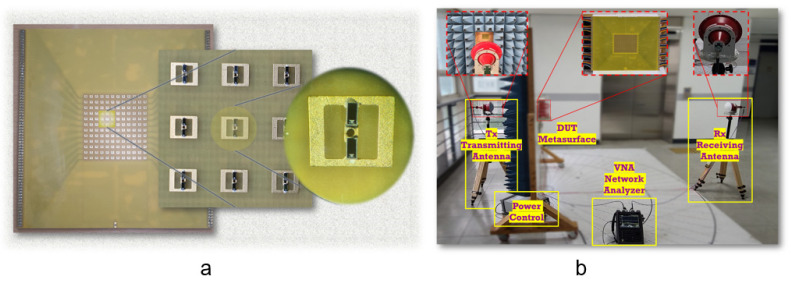
(**a**) Image of the fabricated prototype metasurface with integrated diodes (inset). (**b**) Photograph of the measurement setup.

To validate the simulated results, the proposed MS was fabricated using standard PCB technology. Two layers of the FR4 substrate were fabricated with dimensions of 180 mm × 180 mm (18*λ* × 18*λ*), containing an array of 12 × 12 meta-cells (i.e., 144 elements), as shown in Figure 5a. The two layers were bonded together with an adhesive prepreg layer. The bottom layer contained 288 PIN diodes (two diodes per meta-cell). The state of each meta-cell was selected by the FPGA output pin as either “0” or “1”. Then, the PIN diodes were soldered on the MS using surface mounting technology (SMT) process. Electronic control circuit interfaces were connected to the MS structure through SEMTEC cables. Two power sources were connected to the electronic interface circuit to actuate the switching operations of the transistor circuitry. Two control interfaces (USB-DIO96H modules) with multiple independent output pins were connected to the bias lines through the electronic interface to provide coding sequences to the designed T-DPCM.

### 3.2. Experimental Configuration

The experiments were conducted in an open space, and the experimental configuration for far-field measurements was as shown in Figure 5b. To provide normally incident plane waves, a 26–40 GHz linear-polarised (LP) lens antenna was used as the transmitting antenna, which was located 1 m away from the MS to ensure planarity of the incident wavefront. To measure the resulting radiation from the MS, another LP lens antenna was used as the receiving antenna. Both fabricated samples and the transmitting antennas were mounted on an antenna turntable to provide 360° mechanical rotation in the azimuthal plane. All measurements were obtained with an Agilent Keysight N9951A vector network analyser.

### 3.3. Power Consumption and Switching-Time Calculation

Since the prototype contains 288 PIN diodes (two diodes per meta-cell) where the state of diode is selected using a positive or negative biasing current of 10 mA on each bias line with a voltage drop of 1.3 V, this leads to a total power consumption of about 0.026 W per meta-cell. Thus, the total power consumption of the MS containing 144 meta-cells is 3.744 W.

In our experiment, the PIN diodes were controlled independently in a parallel fashion by an electronic switching circuit and FPGA. The switching/reconfiguration time was determined by the switching time of the PIN diodes, electronic switching transition time, and FPGA clock (CLK) rate.

- The MA4GP907 PIN diode exhibited an exceptionally low RC product of 0.1 ps, and the switching time of the PIN diode was 2 ns.
- The *t*_ON_ time of the NPN BJT (SS8050-G) used in our electronic circuitry with *Vcc* = 3 V and *I*_C_ = 10 mA was *t*_ON_ = *t*_delay_ + *t*_rise_ = (10 + 10) = 20 ns.
- The CLK rate of the FPGA was 100 MHz, which corresponded to the time of each operation cycle of 10 ns.

Since all meta-cells were controlled independently to change PIN diode status in parallel, one operation cycle was needed after compiling. Thus, the total beam re-shaping time was around 32 ns.

### 3.4. Experimental Measurement Results

**Figure 6:**
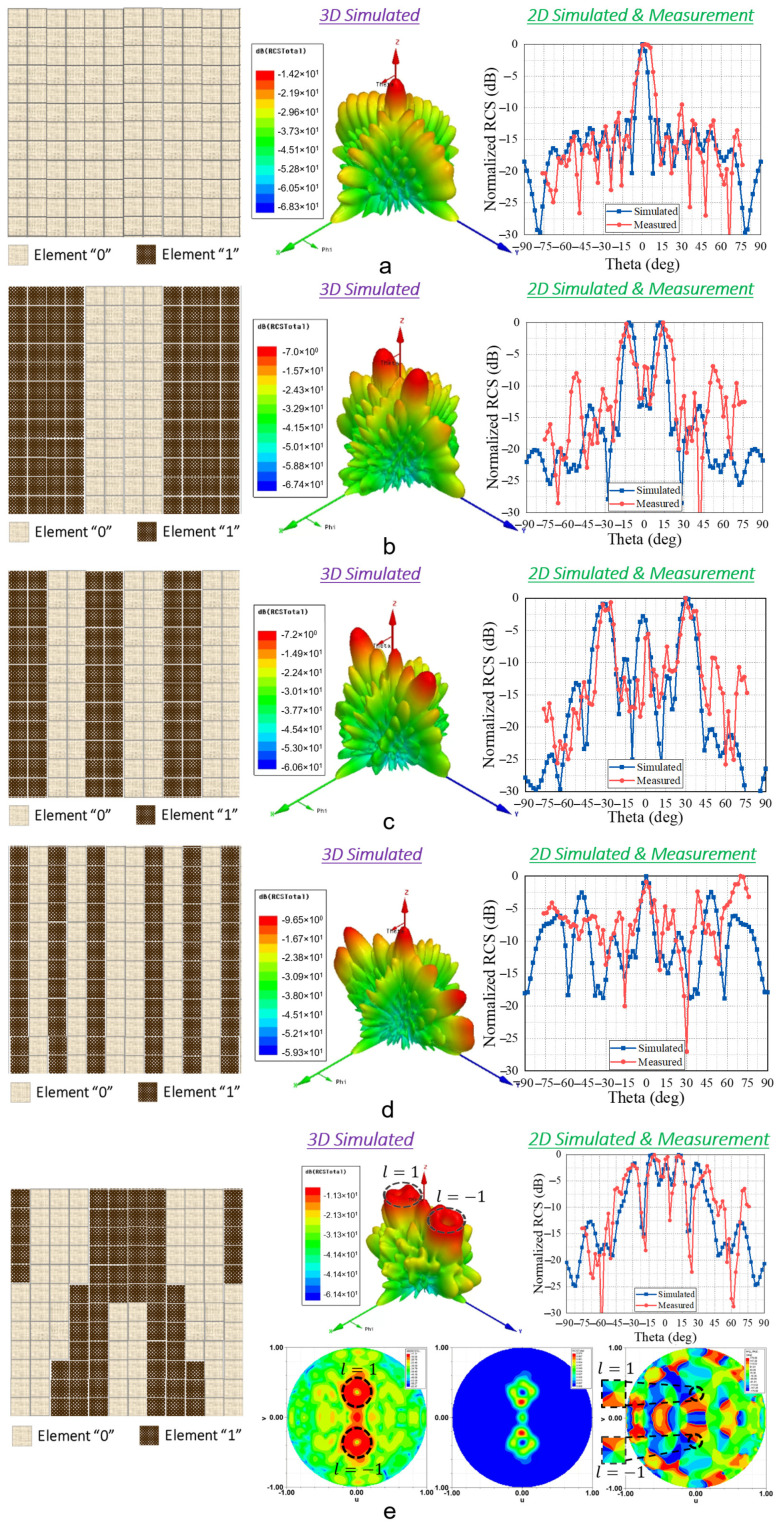
(**a**–**d**) Code distribution, simulated 3D, and 2D-simulated and measured transmitted radiation patterns for different coding sequences. (**e**) Coding sequence for the *l* = ±1 OAM mode with the amplitude and phase distribution plots.

The measured far-field pattern performance of the proposed structure with different coding sequences at 28 GHz is shown in Figure 6a–e. The applied coding sequences were /000000000000/, /111100001111/, /110011001100/, /101010010101/, and OAM dual-beam generation. In the case of dual-beam and multi-beam shaping, the radiated beams at 0° were caused by the phase mismatch between the meta-cell and periodic structure as well as coupling between the adjacent cells. This phase mismatch refers to the sudden change of meta-cell transmission phases in two adjacent 1-bit coding meta-cells which had different states. Their 180° transmission phase difference led to the coupling between the adjacent cells, thus resulting in the side-lobe at 0°. As can be observed from Figure 3 and Figure 6, as the coding sequence periodical length *Γ*_y_ increased, the 0° side-lobe level was significantly reduced due to the better separation between different boundary areas. Besides this, this side-lobe level can also be affected by the transmission magnitude and the number of meta-cells in designed structures. The transmission efficiency was calculated by maintaining the incident power at 1 W. Using periodic boundary conditions, the received power on the receiving side was analysed. The simulated transmission efficiency of the meta-cell was observed to be 51%. Meanwhile, the simulated half-power beamwidth (HPBW) of the proposed design for the broadside case was 9.8°. With reference to Equations (3) and (4), the design of the T-DPCM for maximum steering of the main beam with respect to 3 dB lower than the broadside radiation was investigated via full-wave analysis, and it was observed that the maximum beam-steering/scanning angle was ±42°.

The experimental results contained five transmitted beams for various sequences for dual-beam shaping, i.e., /000000000000/, /111100001111/, /110011001100/, /101010010101/, and OAM dual-beam generation. The experimental and simulated results showed good agreement, and the difference between them was less than 2°. Owing to limitations, the measurement setup was built up to realise dual-beam splitting; however, the quad-beam shaping cases were not measured. In all the test cases of the dual-beam setup, good agreement with the numerical simulations was obtained. On the other hand, Figure 6e illustrates the generation of the OAM dual-vortex beams. The normal incident plane wave was linearly polarised in the *x*-direction with a topological charge of 0. The OAM beams pointing to *θ* = ±20° with opposing topological charges were generated. Two-dimensional simulated and measured transmitted patterns at *φ* = 90° were plotted, where two OAM beams with nulls in the middle were clearly identified. The scattering pattern and phase distribution were symmetrical with respect to the *yoz*-plane, validating the proposed concept.

## 4. Discussion

Table 2 presents a comparison of various reported works in terms of the performance parameters. It should be noted that the number of experimentally reported T-DPCMs in the existing literature is less than the passive designs due to manufacturing challenges. In addition, the experimental T-DPCMs typically focus on the microwave spectrum using diodes (PIN diodes, varactor diodes), as shown in Table 2. Embedding locally tunable elements in the meta-cells becomes more complicated and will increase difficulty when the frequency increases, owing to space limitations caused by the sub-wavelength nature of the meta-cells. However, the proposed design enables a programmable approach through locally tunable PIN diodes in the millimetre-wave frequency band. It was observed that the proposed active design of the T-DPCM has numerous advantages over different reported works in literature in terms of the beam-scanning range, beam-switching time, system power consumption, prototype size, and number of output functions. Another issue with the diode-based designs is that the switching times of commercially available diodes can limit applicability for certain applications. The proposed design has the smallest switching time of 32 ns compared to other active designs. Moreover, a large number of feeding lines makes the design complex, which affects the reliability and performance of the system. The maximum beam-scanning range, which is defined as the maximum steering angle of the main lobe from the boresight direction, was observed to be −73° to +73°, whereas the HPBW for the boresight case was observed to be 9.8°. The simulated transmission efficiency, which is defined as the ratio of receiving power with reference to 1 W incident power for the boresight case, was 51% at 28 GHz.

Table 3 presents the performance comparisons of various reported PCMs based on different techniques and functionalities. Most of the reported works were experimentally investigated at microwave frequencies, whereas some others were demonstrated in the gigahertz and terahertz bands. Although multiple functionalities were achieved (such as beam steering; beam shaping; beam deflection; polarisation control; or advanced functionalities such as vorticity control, OAM beams, and hologram generation), the number of functions on a reported design were typically limited. It is important to note that the novelty of our proposed design lies in the overall system integration, material choice, operating frequency, and the demonstration of specific functionalities through local tuning. These aspects contribute to the uniqueness and novelty of our proposed design. It was observed that when the structure was built using locally tunable elements, where each meta-cell is controlled independently, the T-DPCM had more freedom to incorporate more functionalities, as summarised in the Table 3. However, to date, only one reported T-DPCM in [38] has been investigated and reported with three functions in the terahertz band. It is important to note that owing to the limitations of the measurement setup, only the dual-beam steering case could be experimentally verified; however, the multi-beam, i.e., quad-beam, shaping cases were not measured. The theoretical analysis and experimental results for dual-beam shaping conforms to the proposed idea of achieving various functionalities using different coding patterns.

## 5. Conclusions

This study successfully demonstrated a T-DPCM for Ka-band applications with multi-functional beam-shaping capabilities. Here, the concept of the digital meta-cell comprises binary values “0” and “1”, which represent 0° and 180° phases, respectively. The proposed design enables multi-functionality through locally tunable PIN diodes in the millimetre-wave frequency band. In general, the proposed design has numerous advantages over different reported works in the literature in terms of the beam-scanning range, beam-switching time, system power consumption, prototype size, and number of output functions. In addition, owing to the design completely constructed by low-cost substrates FR-4, the design can significantly enhance the cost effectiveness and thus enable its applicability in practical applications. With the future development of metasurfaces in mind, we foresee programmable metasurfaces as complementary platforms and potential precedents of traditional metasurfaces for a variety of wave manipulation applications.

## Figures and Tables

**Table 1:** Meta-cell detailed parameters in two operational states of 0-bit and 1-bit.

State	Bias Volt.	Diodes State	Trans. Mag.	Trans. Phase	*R*_ON_, *L*_ON_	*R*_OFF_, *C*_OFF_
0-bit	0 V/1.3 V	ON–OFF	−3.4 dB	50.8°	*R*_ON_ = 5.2 Ω *L*_ON_ = 0.05 nH	*R*_OFF_ = 40 kΩ *C*_OFF_ = 0.025 pF
1-bit	1.3 V/0 V	OFF–ON	−4.5 dB	−131.8°		

**Table 2:** Comparison of the designed structure with various reported controllable metasurfaces in the literature.

Ref	Freq. (GHz)	MS Type	Tuning Method	No. of Layers	Dimensions (mm × mm × mm)	Scanning Range (°)	Eff. (%)	Power (W)	Diode Switching Time (ns)	Switching Time (ns)
[23]	8–10	R	PIN-diodes (Global)	2	150 × 150 (4.5λ × 4.5λ)	N/A	N/A	0.4	N/A	N/A
[39]	7.8	R	PIN-diodes (Local)	1	600 × 600 × 2 (15.6λ × 15.6λ × 0.05λ)	N/A	60	0.033	3	33
[40]	5.6–6.1	R	Varactors (Global)	2	260 × 260 × 3.7 (5.1λ × 5.1λ × 0.06λ)	−56.6 to +56.6	79.6	1.6	N/A	N/A
[41]	30	R	Liquid Crystal (Global)	3	50 × 65 × 1.5 (7.02λ × 5.95λ × 0.15λ)	−20 to +20	N/A	N/A	N/A	N/A
[42]	7.1–7.8	T	PIN-diodes (Local)	3	760 × 760 × 3.2 (19.8λ × 19.8λ × 0.08λ)	N/A	29.4	0.2	20	N/A
[38]	408	T	LC (Global)	5	N/A	−30 to +30	50	N/A	N/A	N/A
This work	28	T	PIN-diodes (Local)	3	61.2 × 61.2 × 1.2 (5.7*λ* × 5.7*λ* × 0.11*λ*)	−42 to +42	51	0.026	2	32

**Table 3:** Comparison of the designed structure with various reported coding metasurfaces in the literature.

Ref	Freq. (GHz)	MS Type	Coding Type	Design Approach	Tuning Element	Tuning Mechanism	Functionalities	No. of Functions
[23]	8–10	R	1-bit	Active	PIN-diodes	Global	Beam shaping	1
[43]	28	R	1-bit	Active (analytic)	PIN-diodes	Global	Beam scanning	1
[41]	30	R	2-bit	Active	Liquid crystal (LC)	Global	Beam steering	1
[39]	7.8	R	1-bit	Active	PIN diodes	Local	Holograms	1
[40]	5.6–6.1	R	1-bit	Active	Varactor diodes	Global	Polarisation control	1
[9]	15	R/T	3-bit	Passive	N/A	N/A	Beam deflection, diffuse scattering, vortex beam generation	3
[44]	10	R/T	1-bit	Passive (analytic)	PIN-diodes	Global	Polarisation-dependent OAM beam-steering	1
[42]	7.1–7.8	T	1-bit	Active	PIN-diode	Local	OAM beam generation	1
[38]	0.408	T	1-bit	Active	Liquid crystal (LC)	Global	Dual-beam steering, multi-beam shaping, OAM generation	3
This work	28	T	1-bit	Active	PIN-diodes	Local	Dual-beam steering, multi-beam shaping, OAM dual-beam	3

**Figure A1:**
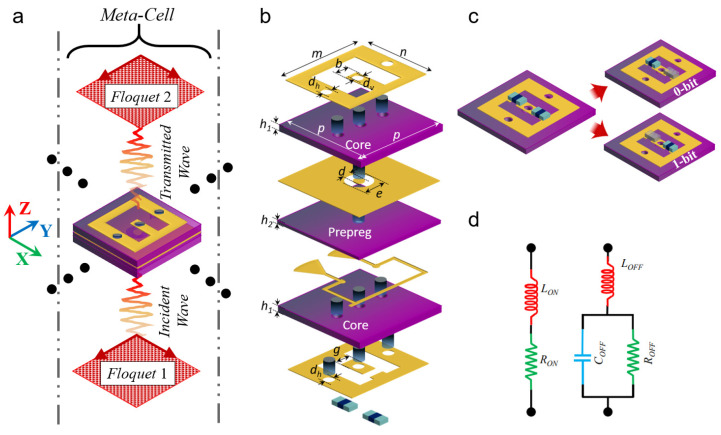
Proposed meta-cell design. (**a**) Perspective view and (**b**) magnified view of the designed meta-cell. (**c**) Bottom receiving layer of the meta-cell with PIN diodes depicted for different meta-cell states of 0-bit and 1-bit. (**d**) Equivalent circuits of the PIN diodes in the ON/OFF states.

## Data Availability

Not applicable.

## Appendix A

We designed and investigated a meta-cell constructed with two layers, namely, the top transmitting layer and bottom receiving layer, fabricated by standard printed-circuit board (PCB) technology using low-cost FR-4 substrates (*ε*_R_ = 4.4, tan*δ* = 0.02) of a thickness of 0.5 mm. These layers were later stacked and manually bonded together with a 0.2 mm thick adhesive prepeg layer (*ε*_R_ = 4.4, tan*δ* = 0.02). On receiving a layer metallic patch, two PIN diodes were integrated. The detailed geometrical dimensions of the designed meta-cells were *d*_v_ = 0.25, *b* = 0.5, *c* = 0.2, *d* = 0.5, *e* = 0.7, *f* = 0.2, *g* = 0.5, *d*_h_ = 0.2, *p* = 5.1, *m* = 2.72, *n* = 2.2, *h*_1_ = 0.5, and *h*_2_ = 0.2 mm.

Because of its low insertion loss and compact size, we chose the PIN diode MA4GP907 as the active tunable device. The diode is modelled as a series RL circuit for forward bias (ON state), i.e., *R*_ON_ = 5.2 Ω; *L*_ON_ = 0.05 nH; and as a shunt RC circuit, i.e., ROFF = 40 kΩ, *C*_OFF_ = 0.025 pF, with reverse bias for the OFF state. The equivalent circuits for the PIN diode in the ON/OFF states of MACOM MA4GP907 are demonstrated in Figure A1.

## References

[1] Cui T., Bai B., Sun H.-B. (2019). Tunable metasurfaces based on active materials. Adv. Funct. Mater..

[2] Awan W.A., Naqvi S.I., Naqvi A.H., Abbas S.M., Zaidi A., Hussain N. (2021). Design and characterization of wideband printed antenna based on DGS for 28 GHz 5G applications. J. Electromagn. Eng. Sci..

[3] Lee J.-G. (2021). Compact and robust Fabry-perot cavity antenna with PEC wall. J. Electromagn. Eng. Sci..

[4] Nkimbeng C.H.S., Wang H., Park I. (2021). Coplanar waveguide-fed bidirectional same-sense circularly polarized metasurface-based antenna. J. Electromagn. Eng. Sci..

[5] Khan H.A., Huang C., Xiao Q., Abbas S.M. (2023). Polarization-dependent coding metasurface with switchable transmission and RCS reduction bands. Micromachines.

[6] Schurig D., Mock J.J., Justice B.J., Cummer S.A., Pendry J.B., Starr A.F., Smith D.R. (2006). Metamaterial electromagnetic cloak at microwave frequencies. Science.

[7] Yang Y., Jing L., Zheng B., Hao R., Yin W., Li E., Soukoulis C.M., Chen H. (2016). Full-polarization 3D metasurface cloak with preserved amplitude and phase. Adv. Mater..

[8] Lian M., Duan L., Chen J., Jia J., Su Y., Cao T. (2022). Acoustic transmissive cloaking with adjustable capacity to the incident direction. Microsyst. Nanoeng..

[9] Zhang L., Wu R.Y., Bai G.D., Wu H.T., Ma Q., Chen X.Q., Cui T.J. (2018). Transmission-reflection-integrated multifunctional coding metasurface for full-space controls of electromagnetic waves. Adv. Funct. Mater..

[10] Fu C., Zhao J., Li F., Li H. (2023). A broadband vortex beam generator based on single-layer hybrid phase-turning metasurface. Micromachines.

[11] Pham D.A., Kim Y., Lim S. (2023). Millimeter-wave coding radial-periodic metasurface for manipulating beam-pointing angle of conical-beam radiation. Wave Random Complex Media..

[12] Larouche S., Tsai Y.-J., Tyler T., Jokerst N.M., Smith D.R. (2012). Infrared metamaterial phase holograms. Nat. Mater..

[13] Huang L., Chen X., Mühlenbernd H., Zhang H., Chen S., Bai B., Tan Q., Jin G., Cheah K.-W., Qiu C.-W. (2013). Three-dimensional optical holography using a plasmonic metasurface. Nat. Commun..

[14] Qiu Y., Chen S., Hou Z., Wang J., Shen J., Li C. (2023). Chiral metasurface for near-field imaging and far-field holography based on deep learning. Micromachines.

[15] Chen W.T., Yang K.-Y., Wang C.-M., Huang Y.-W., Sun G., Chiang I.-D., Liao C.Y., Hsu W.-L., Lin H.T., Sun S. (2014). High-efficiency broadband meta-hologram with polarization-controlled dual images. Nano Lett..

[16] Yuan Y., Wu Q., Burokur S.N., Zhang K. (2023). Chirality-assisted phase metasurface for circular polarization preservation and independent hologram imaging in microwave region. IEEE Trans. Microw. Theory Tech..

[17] Li J., Yuan Y., Yang G., Wu Q., Zhang W., Burokur S.N., Zhang K. (2023). Hybrid dispersion engineering based on chiral metamirror. Laser Photonics Rev..

[18] Ni X., Emani N.K., Kildishev A.V., Boltasseva A., Shalaev V.M. (2012). Broadband light bending with plasmonic nanoantennas. Science.

[19] Sun S., Yang K.-Y., Wang C.-M., Juan T.-K., Chen W.T., Liao C.Y., He Q., Xiao S., Kung W.-T., Guo G.-Y. (2012). High-efficiency broadband anomalous reflection by gradient meta-surfaces. Nano Lett..

[20] Cui T.J., Qi M.Q., Wan X., Zhao J., Cheng Q. (2014). Coding metamaterials, digital metamaterials and programmable metamaterials. Light Sci. Appl..

[21] Di Palma L., Clemente A., Dussopt L., Sauleau R., Potier P., Pouliguen P. (2016). 1-bit reconfigurable unit cell for Ka-band transmitarrays. IEEE Antennas Wirel. Propag. Lett..

[22] Clemente A., Dussopt L., Sauleau R., Potier P., Pouliguen P. (2013). Wideband 400-element electronically reconfigurable transmitarray in X band. IEEE Trans. Antennas Propag..

[23] Wan X., Qi M.Q., Chen T.Y., Cui T.J. (2016). Field-programmable beam reconfiguring based on digitally-controlled coding metasurface. Sci. Rep..

[24] Pan S., Lin M., Xu M., Zhu S., Bian L.-A., Li G. (2022). A low-profile programmable beam scanning holographic array antenna without phase shifters. IEEE Internet Things J..

[25] Tsilipakos O., Tasolamprou A.C., Pitilakis A., Liu F., Wang X., Mirmoosa M.S., Tzarouchis D.C., Abadal S., Taghvaee H., Liaskos C. (2020). Toward intelligent metasurfaces: The progress from globally tunable metasurfaces to software-defined metasurfaces with an embedded network of controllers. Adv. Opt. Mater..

[26] Luo Z., Chen X., Long J., Quarfoth R., Sievenpiper D. (2015). Nonlinear power-dependent impedance surface. IEEE Trans. Antennas Propag..

[27] Wakatsuchi H., Kim S., Rushton J.J., Sievenpiper D.F. (2013). Waveform-dependent absorbing metasurfaces. Phys. Rev. Lett..

[28] Ushikoshi D., Higashiura R., Tachi K., Fathnan A.A., Mahmood S., Takeshita H., Homma H., Akram M.R., Vellucci S., Lee J. (2023). Pulse-driven self-reconfigurable meta-antennas. Nat. Commun..

[29] Pham D.A., Phon R., Kim Y., Lim S. (2021). Batteryless and self-reconfigurable multimode RF network using all-passive energy smart-sensing. IEEE Access.

[30] Pham D.A., Park E., Lee H.L., Lim S. (2021). High gain and wideband metasurfaced magnetoelectric antenna for WiGig applications. IEEE Trans. Antennas Propag..

[31] Pham D.A., Lee M., Lim S. (2021). High-gain conical-beam planar antenna for millimeter-wave drone applications. IEEE Trans. Antennas Propag..

[32] Sarkar A., Pham D.A., Lim S. (2022). 60 GHz electronically tunable leaky-wave antenna based on annular surface plasmon polariton media for continuous azimuth scanning. IEEE Trans. Antennas Propag..

[33] Sarkar A., Pham D.A., Lim S. (2020). Tunable higher order mode-based dual-beam CRLH microstrip leaky-wave antenna for V-band backward–broadside–forward radiation coverage. IEEE Trans. Antennas Propag..

[34] Wang Z., Ge Y., Pu J., Chen X., Li G., Wang Y., Liu K., Zhang H., Chen Z. (2020). 1-bit electronically reconfigurable folded reflectarray antenna based on PIN diodes for wide-angle beam-scanning applications. IEEE Trans. Antennas Propag..

[35] Liu S., Noor A., Du L.L., Zhang L., Xu Q., Luan K., Wang T.Q., Tian Z., Tang W.X., Han J.G. (2016). Anomalous refraction and nondiffractive Bessel-beam generation of terahertz waves through transmission-type coding metasurfaces. ACS Photonics.

[36] Liu S., Cui T.J., Xu Q., Bao D., Du L., Wan X., Tang W.X., Ouyang C., Zhou X.Y., Yuan H. (2016). Anisotropic coding metamaterials and their powerful manipulation of differently polarized terahertz waves. Light Sci. Appl..

[37] Arbabi A., Faraon A. (2017). Fundamental limits of ultrathin metasurfaces. Sci. Rep..

[38] Liu C.X., Yang F., Fu X.J., Wu J.W., Zhang L., Yang J., Cui T.J. (2021). Programmable manipulations of terahertz beams by transmissive digital coding metasurfaces based on liquid crystals. Adv. Opt. Mater..

[39] Li L., Jun Cui T., Ji W., Liu S., Ding J., Wan X., Bo Li Y., Jiang M., Qiu C.-W., Zhang S. (2017). Electromagnetic reprogrammable coding-metasurface holograms. Nat. Commun..

[40] Zhang X.G., Yu Q., Jiang W.X., Sun Y.L., Bai L., Wang Q., Qiu C.-W., Cui T.J. (2020). Polarization-controlled dual-programmable metasurfaces. Adv. Sci..

[41] Wang Q., Zhang X.G., Tian H.W., Jiang W.X., Bao D., Jiang H.L., Luo Z.J., Wu L.T., Cui T.J. (2019). Millimeter-wave digital coding metasurfaces based on nematic liquid crystals. Adv. Theory Simul..

[42] Bai X., Kong F., Sun Y., Wang G., Qian J., Li X., Cao A., He C., Liang X., Jin R. (2020). High-efficiency transmissive programmable metasurface for multimode OAM generation. Adv. Opt. Mater..

[43] Xiao Q., Zhang Y.Z., Iqbal S., Wan X., Cui T.J. Beam scanning at Ka-band by using reflective programmable metasurface. Proceedings of the 2019 International Symposium on Antennas and Propagation (ISAP).

[44] Wu R.Y., Zhang L., Bao L., Wu L.W., Ma Q., Bai G.D., Wu H.T., Cui T.J. (2019). Digital metasurface with phase code and reflection–transmission amplitude code for flexible full-space electromagnetic manipulations. Adv. Opt. Mater..

